# Remarks on the Treatment of Burns by Cold Water

**Published:** 1807-04

**Authors:** Maxwell M'Dowell

**Affiliations:** York, Pennsylvania


### REMARKS *on the* TREATMENT *of* BURNS *by* COLD WATER. *By Dr.* MAXWELL M'DOWELL, *of York, Pennsylvania.*

THE following cases of scalds, which were speedily and completely cured by the application of *cold* water, may perhaps be considered not unworthy of notice. The people, at least in this part of the country, are in possession of so many *infallible* nostrums for scalds and burns, that medical aid is seldom requested in such casualties.

*Case* 1. In the month of January, 1805, my servant girl upset a pot of boiling water upon her right foot; being at home I was early brought to her assistance, but not soon enough to prevent her from taking off her stocking. A complete vesication had taken place over the whole extent of the metatarsal bones, and the stocking removed a piece of the cuticle as large as an half dollar. I immediately had her foot placed in a bucket of cold water, which instantly relieved her of pain. I occasionally dropped a lump of snow into the water, to prevent a diminution of its coldness. I obliged her to sit with her foot in the cold water till she could remove it without experiencing a return of pain. In the course of an hour the cold application was discontinued, and a linen cloth impregnated with ol. oliv. was applied to the denuded cutis. We were not deprived of her services one hour.

*Case* 2. Mrs. ———, about the first of September, 1805, when she was taking some cabbage out of a pot that had just been removed from the fire, overset the pot, and poured the boiling water upon her left foot. Happening to be in her house when the accident took place, I saw her immediately, and, without permitting her to take off her stocking, I had her foot placed in a bucket of cold water. The smarting instantly ceased. In two hours, during which time the water was frequently changed, she was entirely relieved. When the wet stocking was removed the only only discoverable trace of the scald consisted of a slight *blush* of inflammation over the articulation of the toes with the metatarsal bones, which did not prevent her from wearing her shoe. No subsequent application was required.

I have had an opportunity of trying the same remedy in a third case of scald lately, with the like happy result as stated in the above cases.

Since the foregoing cases occurred, I have seen in the Medical and Physical Journal, edited by Professor Barton, a communication from Dr. Thomas Walmsly, in which the Doctor speaks in high terms of the *Tilia Americana* as a remedy for scalds and burns. The Doctor's mode of applying the tilia, is to macerate the "liber" in "cold water" till the water becomes viscid, and keep the part affected "constantly wetted" with it. He considers the cold water as having very little agency in affectiug the cure. The above cases, however, seem to prove, that the Doctor's mode of applying the tilia is not calculated to ascertain its degree of sanative operation in scalds and burns.

Does cold water cure scalds by lessening or suspending the sensibility of the injured part, and also remove the injurious cause, by abstracting the stimulus of heat? My patients were so completely relieved of pain when their feet were placed in cold water, that they considered it unnecessary to let them remain many minutes in it; but when they were indulged in their inclination, they very soon experienced a severe return of smarting.

